# Com-BI-ne: preliminary results of a feasibility trial of brief intervention to improve alcohol consumption and comorbid outcomes in hypertensive or depressed primary care patients

**DOI:** 10.1186/1940-0640-7-S1-A65

**Published:** 2012-10-09

**Authors:** Ruth McGovern, Graeme Wilson, Catherine Wray, Dorothy Newbury-Birch, Elaine McColl, Chris Speed, Anne Crosland, Paul Cassidy, Dave Tomson, Shona Haining, Eileen Kaner

**Affiliations:** 1 Institute of Health and Society, Newcastle University, Newcastle upon Tyne, UK; 2 Department of Pharmacy, Health and Wellbeing, University of Sunderland, Sunderland, UK; 3 Teams Family Practice, Gateshead, UK; 4 Primary Care Mental Health Education and Development Unit, Newcastle upon Tyne, UK; 5 Department of Research and Development, National Health Service North of Tyne, Newcastle, UK

## 

Health outcomes are key to patients and clinicians, but trials of alcohol brief intervention (BI) tend to focus on behavioral outcomes, while those finding positive effects on physical- or mental-health outcomes generally lack power to show robust effects. To test the feasibility of a randomized controlled trial of BI for either hypertensive or depressed primary-care patients who drink alcohol above recommended levels, records at 25 English primary-care practices were searched for adult patients with risky drinking and comorbid hypertension or depression. Eligible patients were randomized to either a hypertension or depression arm, then to a control or intervention condition. They were screened for at-risk drinking using the Alcohol Use Disorders Identification Test (AUDIT). Consenting respondents scoring >7 on the AUDIT completed the Patient Health Questionnaire-9 (PHQ-9) (depression arm) or blood pressure measurement (hypertension arm) and received either BI or standard advice. At six-month follow-up, participants are again screened for alcohol use and comorbid condition. Seventeen practices (median adult patients, 7181; quartile 1, 5195, quartile 3, 8050) searched their databases. Fourteen percent of adult patients (median; quartile 1, 8.4%, quartile 3, 16.5%) drank above guidelines. Twenty percent of adult patients (median; quartile 1, 18.5%, quartile 3, 23.2%) were hypertensive, of which 5% (median; quartile 1, 3.9%, quartile 3, 5.3%) also drank heavily. Fourteen percent of adult patients had mild to moderate depression (median; quartile 1, 8.9%, quartile 3, 16.9%), of which 2% (median; quartile 1, 1.9%, quartile 3, 2.9%) also drank heavily. Of 2590 eligible patients, 633 (24%) completed the AUDIT. Thirty-five percent scored positively on the AUDIT in the hypertension arm, and 50% scored positively in the depression arm. Eighty patients were recruited to the hypertension arm, and recruitment to the depression arm is ongoing. Patients eligible for an RCT of BI for comorbid heavy drinking and hypertension or mild/moderate depression are identifiable in primary care records, though with variation among practices. Almost a quarter of these patients can be screened by mail for current alcohol use; more screen positively in the depression arm than in the hypertension arm. A trial in the hypertension arm seems most feasible.

